# Development of a Wireless Corrosion Detection System for Steel-Framed Structures Using Pulsed Eddy Currents

**DOI:** 10.3390/s21248199

**Published:** 2021-12-08

**Authors:** Namhoon Ha, Han-Seung Lee, Songjun Lee

**Affiliations:** 1 Department of Electrical and Electronic Engineering, Hanyang University, Ansan 15588, Korea; namhoondl@hanyang.ac.kr; 2 Department of Architectural Engineering, Hanyang University, Ansan 15588, Korea; ercleehs@hanyang.ac.kr

**Keywords:** structural health monitoring, wireless sensor network, steel-framed construction, corrosion, pulsed eddy current

## Abstract

Structural health monitoring (SHM) can be more efficient with the application of a wireless sensor network (WSN). However, the hardware that makes up this system should have sufficient performance to sample the data collected from the sensor in real-time situations. High-performance hardware can be used for this purpose, but is not suitable in this application because of its relatively high power consumption, high cost, large size, and so on. In this paper, an optimal remote monitoring system platform for SHM is proposed based on pulsed eddy current (PEC) that is utilized for measuring the corrosion of a steel-framed construction. A circuit to delay the PEC response based on the resistance–inductance–capacitance (RLC) combination was designed for data sampling to utilize the conventional hardware of WSN for SHM, and this approach was verified by simulations and experiments. Especially, the importance of configuring sensing modules and the WSN for remote monitoring were studied, and the PEC responses caused by the corrosion of a specimen made with steel were able to be sampled remotely using the proposed system. Therefore, we present a remote SHM system platform for diagnosing the corrosion condition of a building with a steel structure, and proving its viability with experiments.

## 1. Introduction

SHM, which evaluates the durability of building structures, diagnoses points with damage and finds their location by collecting data using a sensor system in real time [1]. Additionally, advanced methods were introduced to reconstruct the lost data for the precise SHM. [2,3]. However, the conventional wired system used in SHM is uneconomical because a large amount of wire is necessary and requires substantial labor during the installation and maintenance periods. If a WSN is applied to the SHM system, building structures can be conveniently maintained with a low cost [4,5]. When considering the total economic costs of WSN SHM, the operating time should be taken into account, because the WSN is generally powered by a battery.

SHM includes measuring temperature, humidity, wind speed, earthquake incidence, and corrosion. Corrosion, which affects the durability of buildings, occurs in all steel structural materials. Various steel sections, such as wide flanges, I-beams, and channels, are used for structures such as buildings, roads, and bridges, which can be easily seen around us, and these structures are classified by the following construction method. Steel-framed construction (SC) [6] is constructed quickly and has low local environmental pollution and can be applied for the construction of building parking lots, but, in this case, severe corrosion can occur due to direct exposure to weather conditions [7]. Steel-framed reinforced concrete (SRC) has a light self-weight, high strength, and high stiffness by combining steel beams with reinforced concrete (RC) [8] and is used in large construction projects, such as skyscrapers, bridges, and tunnels. However, bridges are easily corroded by ionic deicing chemicals used in winter [9], and subsea tunnels are also corroded due to electrochemical action caused by chloride ion invasion [10]. The corrosion of steel-framed construction, which is caused by various factors in the diverse environments, should be measured to provide a warning before breakdown, because it can cause cracks, the spalling of concrete, and structural collapse [11].

**Figure 1:**
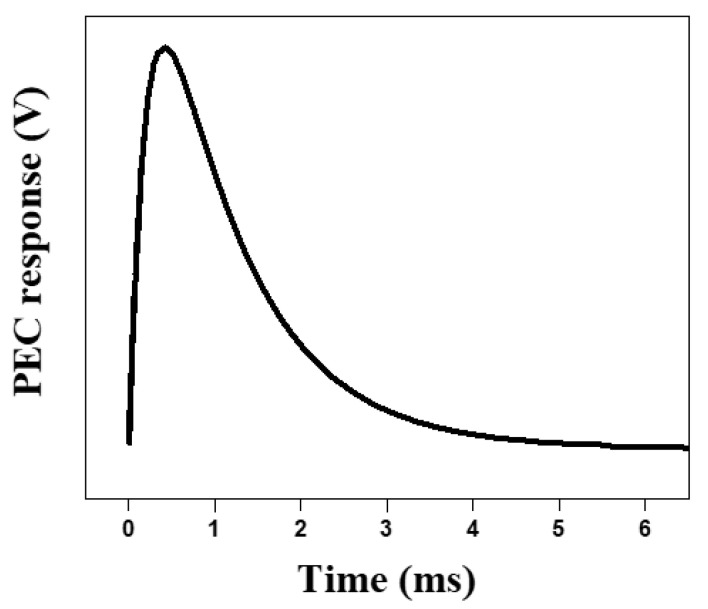
A typical PEC response.

There are several technologies used to measure corrosion, including the following methods: eddy current [12,13], ground penetrating radar (GPR) [14], galvanostatic pulse method (GPM) [15], fiber Bragg grating (FBG) [16], ultrasonic pulse velocity (UPV) [17], and infrared thermography (IRT) [18]. Eddy current testing (ECT) is the method used in this research. The mechanism of the eddy current method is to measure the conductivity and permeability changes caused by corrosion by inducing an eddy current that is generated by a sine wave or pulse [19,20,21,22,23]. Although the method using a single-wavelength sine wave successfully measures corrosion, the detectable depth is limited by the skin effect. Since corrosion occurs not only on the surface of a steel frame but also inside of it, it is appropriate to use multiple-frequency waves as input signals to evaluate the durability of structures. However, the method of generating multiple frequencies requires much more complex and expensive electronics than a single-frequency system, involving generating input signals and measuring output signals, and it is not suitable for minimizing cost or power consumption. On the other hand, the method of PEC using a pulse signal as an input has the possibility to minimize the power consumption [24,25]. Further, since it covers multiple frequencies which can detect various depths of objects without actually changing the frequency [12,26], PEC can be used as an alternative to explicit multiple-frequency methods for inspecting corrosion deep inside a steel frame. Thus, to detect corrosion, the pulse input is applied for the steel structure, and the output from the PEC response should be measured. A typical PEC response appears as an exponential decay for several milliseconds [27], shown in Figure 1. Data acquisition (DAQ) equipment, such as an analog-to-digital converter (ADC) board, is used to measure the response [28,29], but this configuration is unsuitable for application in an actual situation that requires a low-power system, such as the typical WSN environment. For a WSN, the low cost, small size, and low power consumption are usually taken into account. Therefore, measuring the PEC response is difficult when applying a conventional system for use in an actual SHM application. 

In this paper, we propose a method for detecting the corrosion of a steel-framed construction with a convenient monitoring system using WSN. A circuit designed to delay the PEC response makes it possible to easily deploy in an actual construction environment. After a PEC is induced to detect corrosion, in order to measure the response, a delay circuit for the response signal should be provided, because the output signals vary too fast for sampling them using a conventional measurement system. By applying the proposed method using the WSN, the remote monitoring system can be utilized for a more convenient real-time analysis of the corrosion state. In order to achieve this, tiny sensor modules, without large-sized, general-purpose measuring equipment, should be developed and installed in various locations of the building for a more efficient and precise SHM. Additionally, the more collected data from the several parts of a building, the more efficient SHM is. Additionally, various analysis environments with a convenient user interface are provided in the hardware configurations.

## 2. Pulsed Eddy Current Response

PEC response must be measured to evaluate the amount of corrosion, but it is difficult to measure this with the hardware configuration generally used for WSNs. (Figure 1) In this section, a delay circuit designed to sample PEC responses, in order to compare the results from the hardware configuration of a conventional WSN system, is described. 

**Figure 2:**
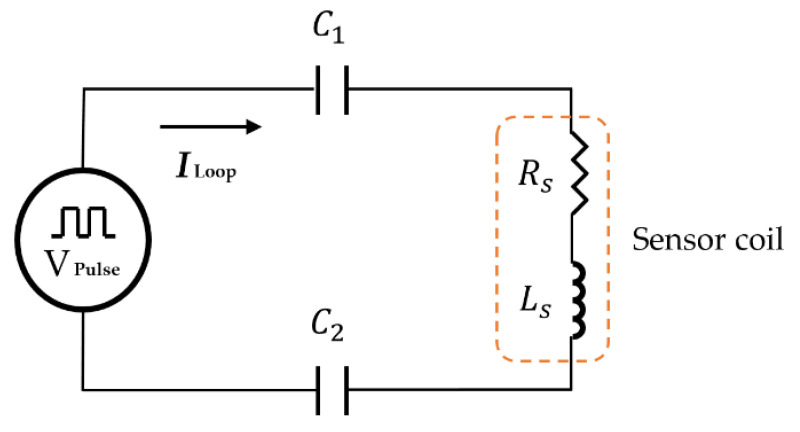
The delay circuit of a sensor module for sampling PEC response.

Figure 2 shows the configuration of the proposed delay circuit of the sensor module. It is based on an RLC circuit and constitutes a loop circuit by connecting all components in series. $C_{1}$ and $C_{2}$ are capacitors, and a sensor coil is placed between them. The sensor coil has an inductance ($L_{s})$ and resistance ($R_{s}$). $V_{Pulse}$ is the input source, and $I_{Loop}$ is the current flowing in the loop circuit. According to Kirchhoff’s voltage law (KVL), the circuit is described as follows:

(1) $$V_{Pulse}+\frac{j}{\omega C_{1}}I_{Loop}-\left(R_{S}+j\omega L_{S}\right)I_{Loop}+\frac{j}{\omega C_{2}}I_{Loop}=0_{\;}$$

However, Equation (1) is based on the circuit in Figure 2 without any specimen. Therefore, when measuring corrosion, the effective resistance of the coil sensor ($\Delta R_{V}$) is varied by the specimen. It is defined as follows:

(2) $$R_{VS}=R_{S}+\Delta R_{V}$$

The effective inductance of the sensor ($\Delta L_{V}$) coil is also altered by the specimen, which could be a conductive material, and is defined as follows:

(3) $$L_{VS}=L_{S}+\Delta L_{V}$$

The substitution of Equations (2) and (3) into (1) is expressed as follows:

(4) $$V_{Pulse}+\frac{j}{\omega C_{1}}I_{Loop}-\left(R_{VS}+j\omega L_{VS}\right)I_{Loop}+\frac{j}{\omega C_{2}}I_{Loop}=0$$

$I_{Loop}$ is calculated as Equation (5):

(5) $$I_{Loop}=-\frac{V_{Pulse}}{j\left(\frac{1}{\omega C_{1}}+\frac{1}{\omega C_{2}}-\omega L_{VS}\right)-\left(R_{VS}\right)}$$

The voltage of $C_{2}$ is expressed as the following Equation (6):

(6) $$V_{C2}=\;-j\frac{I_{Loop}}{\omega C_{2}}$$

Using Equations (5) and (6) can be described as follows:

(7) $$V_{C2}=\;j\frac{V_{Pulse}}{\omega C_{2}\left[j\left(\frac{1}{\omega C_{1}}+\frac{1}{\omega C_{2}}-\omega L_{VS}\right)-\left(R_{VS}\right)\right]}$$

Therefore, it can be seen that a change in the specimen’s electrical properties produces a variation in the PEC. 

**Figure 3:**
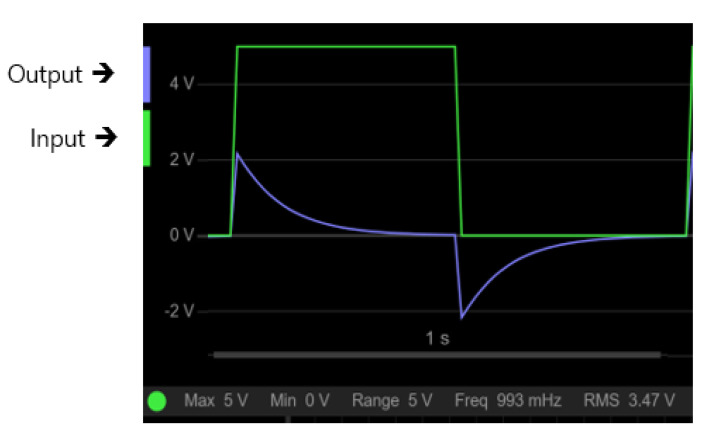
A simulation of the delay circuit for monitoring the PEC response.

In order to operate the delay circuit for a simulation, 4.7 nF ceramic capacitors are selected for both $C_{1}$ and $C_{2}$, and the inductance and resistance of the sensor coil are set to 195 uH and 2.5 Ω, respectively. The $V_{Pulse}$ square wave with 1 Hz, is an input to the RLC circuit. Figure 3 shows the result achieved by an electronic circuit simulator, EveryCircuit, confirming that the PEC response is delayed by the proposed circuit. The exponentially decaying signal obtained by simulation appeared for about 500 ms, and demonstrated that it took approximately 100 times longer than the general PEC response shown in Figure 1.

## 3. Corrosion Remote Monitoring System

In this section, we propose a system for implementing the design described in the previous section, which is able to sample the delayed PEC response. The sensor node is made by combining the proposed circuit with the hardware configuration of a conventional Zigbee-based WSN for remote monitoring. Additionally, a user-friendly interface is implemented for convenience. 

### 3.1. Sensor Node Design

**Figure 4:**
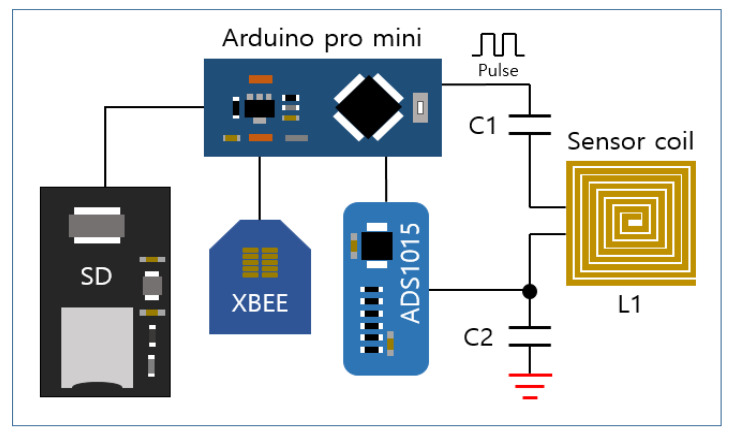
Hardware configuration of the sensor node.

For the detection and remote monitoring of corrosion, the proposed system requires several functions, including data collection and communication. Figure 4 shows the hardware configuration of the sensor node. An Arduino-based system, which has many commercial modules for expansion, sufficient open library codes, and a high compatibility, provides a complimentary integrated development environment (IDE) for developers. Thus, the Arduino Pro Mini was chosen to develop the hardware for the proposed system. In addition, it is utilized as a pulse generator, because a digital input signal can be generated by controlling its GPIO. On the other hand, in the case of a conventional ECT, a magnetic sensor, such as a Hall sensor, is used to detect the intensity of the eddy current, but, in this research, the only variation of the voltage of the coil caused by the PEC is measured without any additional sensor, while the PEC is induced on a specimen through the same sensor coil. If the number of turns of the coil for the sensor is increased, the intensity of PEC response becomes high. Thus, the sensor coil with a larger size has a higher sensitivity. In order to determine the size of the sensor, it is designed according to the size and shape of the part that is measured, optimized by a simulation or experiment, and then applied to the actual building structures. In this research, the sensor coil has a planar square shape. It can be placed close to a steel component, and is suitable for measuring the corrosion of a large area. In order to make the planar coil sensor, a foam board, double-sided adhesive tape, and AWG26 wire were used, and its size is arbitrarily chosen to be 150 mm by 150 mm for an experiment. For sampling the PEC response signals detected by the sensor coil, the ADS1015 analog-to-digital converter is used, which has a 12-bit resolution and a sampling rate of 3300 samples per second. The sampled data are stored in an SD card for every sampling as backup data, which can prevent data loss caused by communication errors. In this research, a sampling rate of 500 samples per second is chosen for the experiment, and a smaller sampling rate is possible to detect corrosion for SHM. The proposed system configures the WSN to remotely transmit the measured data to the master node of a Zigbee communication device (xBee s2c, DIGI, Hopkins, MN, USA). The Zigbee module based on the IEEE 802.15.4 standard has various advantages such as a low power consumption, low cost, and high compatibility with various network topologies. Therefore, it is suitable for an actual application of a WSN for structural health monitoring.

### 3.2. Networking and Monitoring

**Figure 5:**
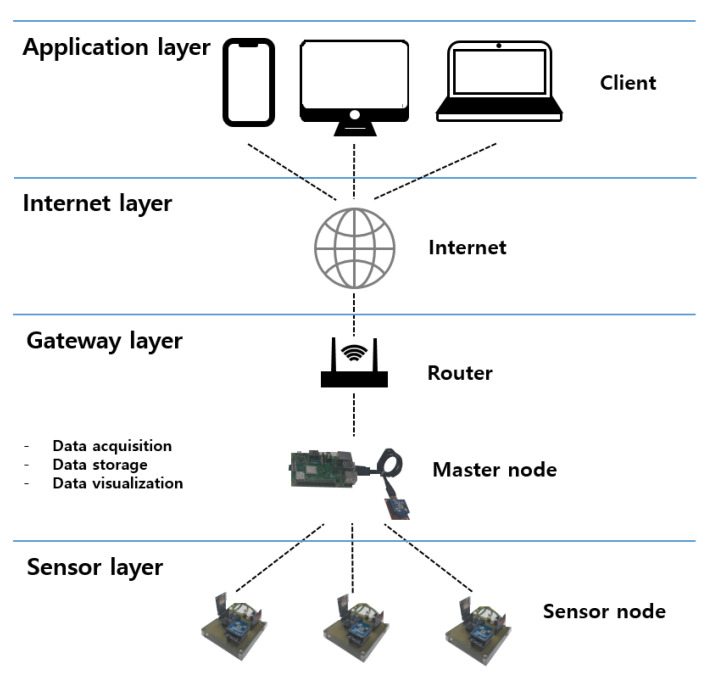
The proposed architecture of the monitoring system.

Figure 5 shows the architecture of the system for remotely monitoring the corrosion data collected from the sensor node. The master node in the gateway layer is implemented using a Raspberry Pi 3 B+, which is a single-board computer the size of a credit card and has a built-in operating system. It is used as both a master node and server. In order to use a relational database management system, MariaDB is installed to collect data from the sensor node and for storage in the database. The data are visualized for analysis, and Grafana, a web-based interactive application, provides convenient features to visualize the data from a database. Additionally, the Raspberry Pi has both an ethernet port and a wireless LAN, and it can be connected to the Internet without an additional device. As a result, users can access the master node through the Internet and monitor the data measured from the construction site anytime and anywhere. 

## 4. Experimental Section

**Figure 6:**
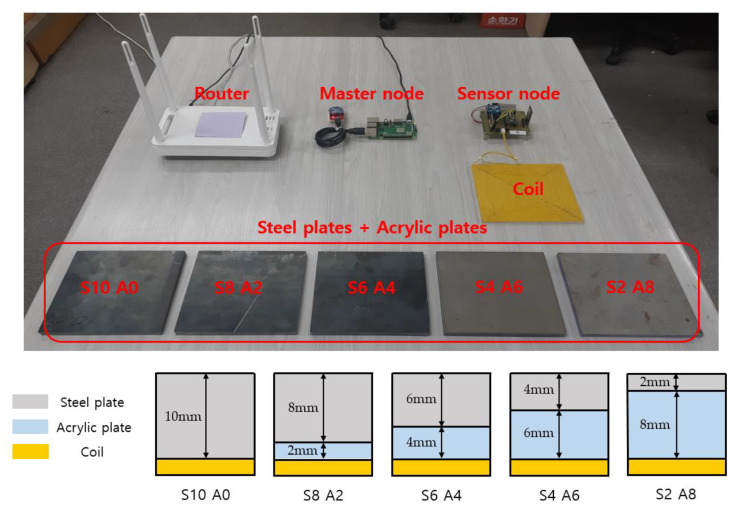
The experimental setup.

Experiments were conducted to confirm the delay in the PEC response and verify the detectability of corrosion of the steel plate. Figure 6 shows the experimental setup to examine corrosion using the proposed system. The lift-off effect is caused by variations in the distance between the sensor and the specimen. The thickness of the rust on the specimen is regarded as the variation of distance, and it becomes a factor that interferes with the signal that is collected. Several studies aimed to reduce this effect [30,31,32]. However, when the sensor coil is buried or fixed, the effect is generated by the corrosion of the specimen, and the same principle of a thickness measurement is applied to the evaluation of corrosion [13]. Accordingly, in the experimental setup, the thickness reduction due to corrosion is replaced by the change in the thickness of the steel plates, and the consequent lift-off effect was simulated by placing non-conductive acrylic plates between the steel plate and the coil. The size of the steel plates and the acrylic plates used in the experiment were each 150 mm by 150 mm, and had the same dimensions as the sensor coil with thicknesses of 2 mm, 4 mm, 6 mm, 8 mm, and 10 mm. The collected experimental data were recorded and monitored by the proposed system. This experiment measured the change in the response of PEC according to the thickness of corrosion. When installed in an actual building, the sensor coil should not prevent the corrosion of the steel frame, so it should be installed at a suitable distance.

## 5. Result

**Figure 7:**
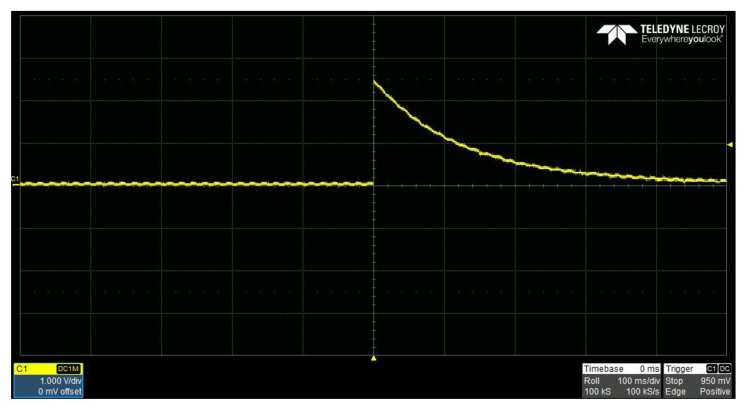
The delayed PEC response measured by the sensor circuit.

In order to verify the delay of the PEC response using the sensor circuit explained in Section 2, the signal was measured using an oscilloscope. Figure 7 shows the output signal when the pulse is applied to the sensor circuit. The signal induced by the proposed sensor circuit showed a peak value of approximately 2.5 V and an attenuation of a signal of about 500 ms, which were very similar to the simulation result in Figure 3. It shows that the sampling is possible by the ADC, which is generally used in the actual situation.

**Figure 8:**
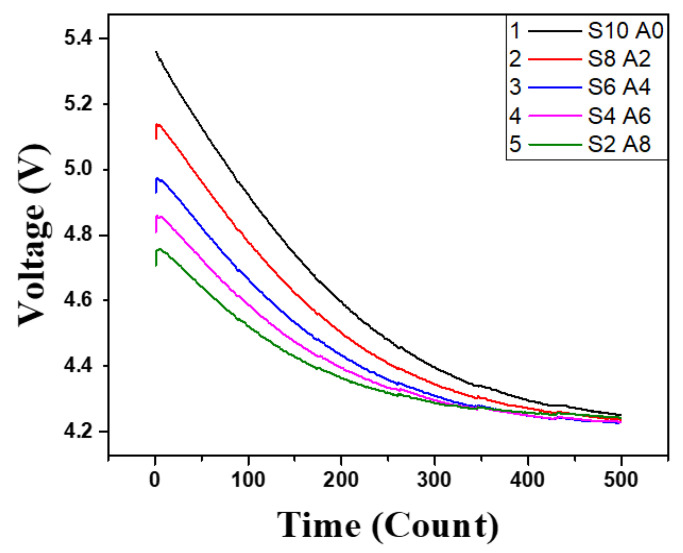
The variation of the PEC response by corrosion level.

Figure 8 shows the experimental result of sampling the delayed PEC response using the proposed system and examines if corrosion is detected from the steel-framed construction sample. Each line in Figure 8 indicates the voltage variation of $V_{C2}$ according to the level of corrosion, which represents the PEC response that is sampled 500 times for about one second by the sensor node. The thicknesses of the steel plates (S) and the acrylic plates (A) used to simulate the depth of corrosion were 10 mm for the steel plate and 0 mm for the acrylic plate for Line 1; 8 mm for the steel plate and 2 mm for the acrylic plate for Line 2; 6 mm for the steel plate and 4 mm for the acrylic plate for Line 3; 4 mm for the steel plate and 6 mm for the acrylic plate for Line 4; and 2 mm for the steel plate and 8 mm for the acrylic plate for Line 5, respectively. According to the results, the delayed PEC response was successfully sampled by the sensor node with the proposed circuit, and the corrosion was identified by considering the fact that the peak value of $V_{C2}$ consistently decreased as the corrosion progressed. The significant thickness of corrosion depends on the kinds of building structures and their environments [33]. The amount of corrosion can be evaluated without a high-end data acquisition device nor a signal conditioner with a computer system, and the cost of the sensor module can be reduced because the coil is utilized for both inducing eddy current and detecting the effect of the eddy current without an additional device. Thus, the developed low-cost wireless SHM system can be utilized to conveniently measure the corrosion of steel-framed construction with convenience at an actual construction site. In addition, for the precise SHM, the simulation or experiment for the specific condition should be carried out before installing the system for an actual building structure. In case of the long-term stability of the system, the accumulated measurement error of sensor inside the building structure, due to various causes, should be corrected by periodic calibration so that it is not regarded as corrosion.

**Figure 9:**
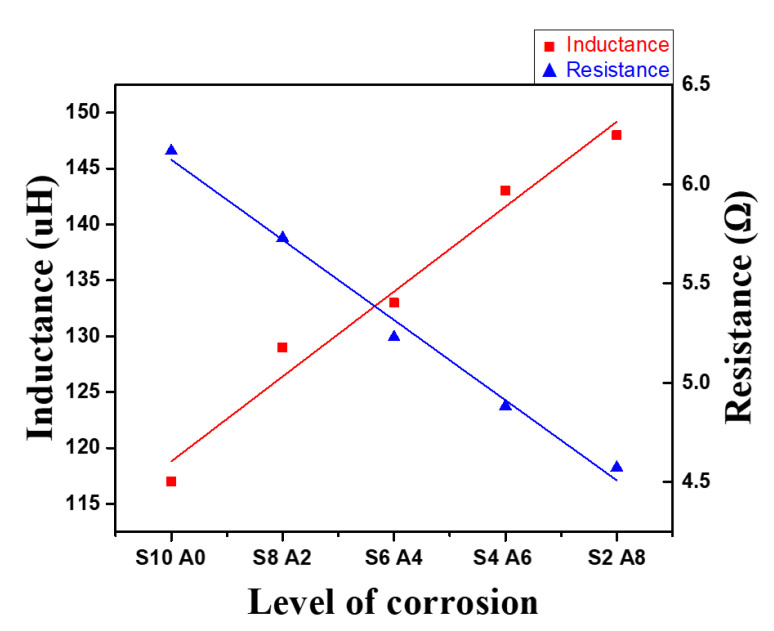
Resistance and inductance of the sensor coil by corrosion level.

Additionally, the variations of the inductance and the resistance of the sensor coil were examined using an LCR meter to verify the relationship with the corrosion level. (Figure 9) The red line indicates the inductance of the sensor coil, and the blue line indicates the resistance of sensor coil. As a result, the inductance and resistance varied by up to 26%, and 35%, respectively, due to the corrosion level. The PEC induced in the steel plate generates a magnetic field whose direction is opposite to that of the sensor coil. The inductance of the sensor coil decreases due to the reduction in magnetic flux in the coil. Additionally, the resistance of the sensor coil increases because of the energy dissipation caused by the PEC [34]. This was verified by experiments and analyses in the study.

**Figure 10:**
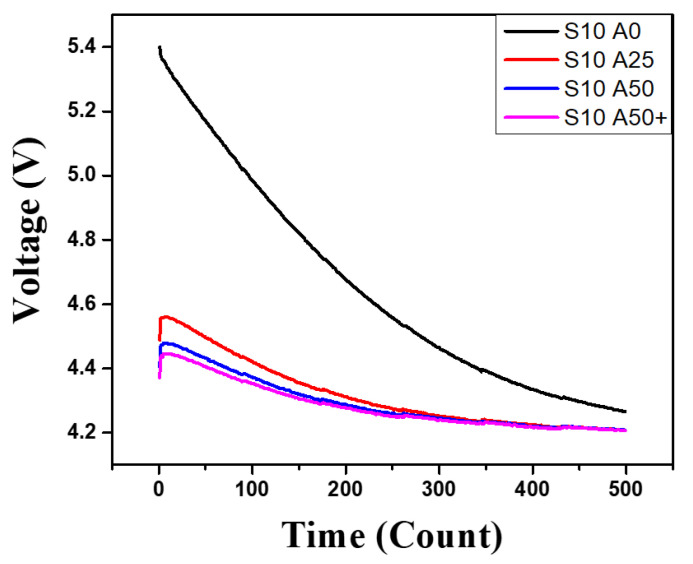
Detectable range of the proposed sensor.

Figure 10 shows the detectable range of the sensor, which is experimental result measured using a steel plate and acrylic plates (0 mm, 25 mm, 50 mm). According to the blue line (S10 A50), the maximum distance for the detection of steel plate is about 50 mm because it is almost similar to the pink line (S10 A50+), which shows the voltage values in the case that the steel plate is placed further than 50mm from the sensor coil.

In the experiments, a viability of applying the proposed method to a steel-framed structure was confirmed by the fact that the corrosion level was detected by variation of PEC. According to the previous work [13], detecting the corrosion of building structures is possible using PEC methodology. Tiny sensor modules developed by the proposed method can be installed in various parts of the real building structures more easily than conventional hardware equipment for SHM.

**Figure 11:**
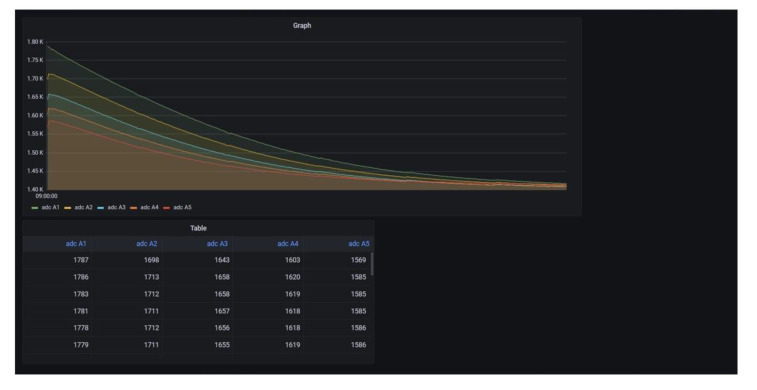
The dashboard for monitoring corrosion.

Figure 11 is the screen that shows the results, according to corrosion status, using the Grafana web-based monitoring application, based on the hardware configuration explained in Section 3. Grafana provides various visualization tools, such as graphs, tables, and bar charts, which use data from the sensor nodes. Moreover, the server computer, implemented with a Raspberry Pi, is suitable for the proposed system because of its low cost and tiny size. Therefore, users can easily access the monitoring system without the restrictions of time and space, provided that they have a smartphone or tablet PC connected to the Internet. 

## 6. Conclusions

In this paper, we proposed a platform that could perform the remote monitoring of the corrosion of steel-framed construction in real time using the PEC method. For the SHM of the steel-framed construction, the PEC response was sampled with the designed hardware configuration containing a delay circuit, and the performance of the sensor was confirmed by measuring the corrosion of the test sample in experiments. Furthermore, by applying WSN and IoT technology, a real-time remote monitoring system was implemented that was easily accessible for user and could efficiently analyze the status of corrosion with database and visualization software. By using the proposed method, the tiny, low-cost hardware module for SHM can be manufactured without a function generator, oscilloscope, and other general-purpose measuring equipment. Additionally, real-time and remote monitoring become possible if they are applied to many locations in the building structure with wireless networks. Thus, the efficiency becomes higher than the conventional SHM method. Moreover, the system also provides convenience, efficiency, and portability by using a Raspberry Pi for the server computer.

Furthermore, the proposed system can be applied in other fields, such as non-destructive testing related to cracks or the thickness of paint for aircraft and ships by designing sensor coils with various shapes depending on the particular desired application.

The limitation of the proposed system is the power consumption for the long-term measurements. The power consumption of sensor modules can be reduced by data compression and more efficient communication algorithms, etc. Additionally, the operation time can be improved by applying energy harvesting techniques such as wind and solar power, etc.

## Data Availability

Not applicable.
